# Induced Pluripotent Stem Cells: Challenges and Opportunities for Cancer Immunotherapy

**DOI:** 10.3389/fimmu.2014.00176

**Published:** 2014-04-17

**Authors:** Patty Sachamitr, Simon Hackett, Paul Jonathan Fairchild

**Affiliations:** ^1^Sir William Dunn School of Pathology, University of Oxford, Oxford, UK

**Keywords:** cancer, immunotherapy, dendritic cell, cytotoxic T cell, NK cell, pluripotency, iPS cell

## Abstract

Despite recent advances in cancer treatment over the past 30 years, therapeutic options remain limited and do not always offer a cure for malignancy. Given that tumor-associated antigens (TAA) are, by definition, self-proteins, the need to productively engage autoreactive T cells remains at the heart of strategies for cancer immunotherapy. These have traditionally focused on the administration of autologous monocyte-derived dendritic cells (moDC) pulsed with TAA, or the *ex vivo* expansion and adoptive transfer of tumor-infiltrating lymphocytes (TIL) as a source of TAA-specific cytotoxic T cells (CTL). Although such approaches have shown some efficacy, success has been limited by the poor capacity of moDC to cross present exogenous TAA to the CD8^+^ T-cell repertoire and the potential for exhaustion of CTL expanded *ex vivo*. Recent advances in induced pluripotency offer opportunities to generate patient-specific stem cell lines with the potential to differentiate *in vitro* into cell types whose properties may help address these issues. Here, we review recent success in the differentiation of NK cells from human induced pluripotent stem (iPS) cells as well as minor subsets of dendritic cells (DCs) with therapeutic potential, including CD141^+^XCR1^+^ DC, capable of cross presenting TAA to naïve CD8^+^ T cells. Furthermore, we review recent progress in the use of TIL as the starting material for the derivation of iPSC lines, thereby capturing their antigen specificity in a self-renewing stem cell line, from which potentially unlimited numbers of naïve TAA-specific T cells may be differentiated, free of the risks of exhaustion.

## Cancer and the Immune System: A Historical Perspective

Although it was Paul Ehrlich who, in 1909, first introduced the concept of the immune system as a means of controlling the incidence of cancer, it was 50 years later, with development of the field of cellular immunology and discovery of the role of the immune system in allograft rejection (1), that this notion first gained traction. In 1970, Burnet and Thomas introduced the concept of immunological surveillance and postulated that the immune system had a mechanism for eliminating potentially dangerous mutated cells and speculated that lymphocytes were actively involved in the process by the recognition of neo-antigens, either unique to the tumor (tumor-specific antigens; TSAs) or shared by other somatic cells (tumor-associated antigens; TAAs) (2, 3). This theory was met with skepticism, due in part, to the observation that the incidence of tumors in immune compromised *nude* mice did not differ substantially from their wild type counterparts (4, 5). These observations were, however, counter-balanced by the discovery that tumors may lack immunogenicity, not due to the absence of TAAs *per se* but rather their inability to activate the immune system (6). This subsequently gave rise to the modified concept of cancer immunoediting (7), which postulates that a developing tumor is under a constant immunological selection pressure, leading either to its elimination, the establishment of a dynamic equilibrium between the tumor and the immune system, or its escape from immune surveillance, resulting in unopposed growth. It is now accepted that one of the hallmarks of cancer is the lack of immune regulation (8) and that certain cancers therefore have the propensity to induce a state of autoimmunity in some individuals. While the underlying mechanisms remain to be clarified, mutations in specific TAAs may increase their immunogenicity, eliciting T and B cell responses that readily cross-react with the wild type protein, expressed in other cell types or anatomical locations. Indeed, a recent study has shown that certain cancer patients develop the chronic autoimmune rheumatic disease, systemic sclerosis (9), illustrating the important concept that immune surveillance harnesses elements of the autoreactive T-cell repertoire to elicit anti-tumor responses, sometimes at the cost of collateral damage to self-tissues: it is the same autoreactive repertoire that cancer immunotherapy seeks to recruit.

## Approaches to Cancer Immunotherapy

Radiation, chemotherapy, and surgery are the three traditional methods for controlling the spread of cancer, which, although effective, may fail to completely eliminate neoplastic cells or the cancer stem cells that sustain a developing tumor. Additionally, the lack of specificity of these approaches and the damage to otherwise healthy tissues may lead to severe morbidity and, in extreme cases, mortality. Given its inherent specificity, adaptability, and capacity to generate a memory response, cancer immunotherapy promises to be more effective and durable than classical treatment modalities (10).

Cytokines, such as interleukin-2 (IL-2), interferon-α (IFN-α), and tumor necrosis factor-α (TNF-α) have been used non-specifically to stimulate an anti-tumor response. These cytokines act either by directly inhibiting growth of the tumor cells or by promoting proliferation and sustained cytokine production by T cells and NK cells, thereby increasing their ability to target tumor cells. Some cytokines, such as granulocyte-macrophage colony-stimulating factor (GM-CSF), act on antigen-presenting cells (APCs), inducing upregulation of MHC and co-stimulatory molecules, which promote their capacity to activate lymphocytes. A number of cytokines, used singly or in combination, have proven effective in increasing the anti-tumor immune response and have, in recent years, entered clinical trials for the treatment of advanced cancer (11). IL-2 has, for instance, been approved by the US Food and Drug Administration (FDA) for the treatment of metastatic melanoma and renal-cell carcinoma (12). However, given the non-specificity of the approach, low response rates, and toxic side effects, additional understanding of cytokine signaling pathways and their function *in vivo* are still required (11).

The identification of a number of well-defined TAAs and Tissue Specific Antigens (TSAs), along with the development of hybridoma technology (13), has facilitated the production of monoclonal antibodies (mAbs) that either directly target these antigens or block central pathways involved in tumor function. mAbs have, for instance, been used to inhibit molecules such as CTLA-4 and PD-1, since upregulation of their ligands by tumor cells may inhibit T-cell function, enhancing their ability to evade immune surveillance (14, 15). In recent clinical trials of the PD-1-specific mAb, BMS-936558, objective and durable responses were observed in approximately one in four to one in five patients with non-small cell lung cancer, melanoma, or renal-cell cancer. Immunohistochemical staining of tumor specimens prior to the onset of treatment, revealed that intra-tumoral expression of PD-1 ligand (PD-L1) correlated with the induction of anti-tumor responses, providing a means of stratifying patients in order to identify those most likely to respond to treatment and greatly increasing the likely future clinical impact of mAb therapy (16). Although it was the exquisite specificity of mAbs that first earned them the name “magic bullets,” response rates remain disappointingly low when used as a single therapy (17–19). One reason for this poor performance may lie in the fact that administration of therapeutic mAbs is inherently passive, failing to generate a memory response. Furthermore, the repeated administration required as a result, may elicit neutralizing anti-idiotypic responses, which greatly limit efficacy.

In contrast, the ultimate aim of cancer immunotherapy is to activate the immune system to recognize the tumor, thereby generating a specific and durable effector T-cell response. In order to achieve this goal, adoptive transfer of TAA-specific T cells has been explored, involving their expansion *ex vivo* and re-administration to the same patients from whom they were originally derived (20). Alternatively, the ability of DCs to present TAAs to T cells has been harnessed to generate an immune response against tumor cells. Whereas adoptive T-cell transfer may generate a burst of T-cell immunity that is short-lived, DC-based vaccines have the potential to induce a sustained immune response with the capacity for subsequent recall (21). It has been shown that DC vaccination, following adoptive T-cell transfer, may further boost anti-tumor responses, suggesting a rationale for combining the two therapeutic strategies (21). In this review, we shall discuss recent experience of harnessing DCs and T cells for cancer immunotherapy and obstacles hindering their success. We shall also focus on the emerging use of patient-specific induced pluripotent stem cells (iPSCs) for the differentiation of DCs, T cells, and NK cells and discuss how this novel source holds promise for overcoming some of the shortfalls of conventional cancer immunotherapy.

## Cell-Based Immunotherapies and Their Obstacles

As sentinels of the immune system, DCs play a pivotal role in eliciting the primary immune response to antigen. The ability of DCs to process and present protein antigens via the canonical endocytic pathway is crucial to this process (22). However, some subsets of DCs also possess the ability to capture exogenous antigens and cross present them via MHC class I direct to CD8^+^ T cells, thereby eliciting a cytotoxic T-cell response (23–25). Since cancer cells are poor APCs due to constitutively low expression of MHC class I and II determinants, the generation of protective anti-tumor immunity depends upon the cross presentation of tumor antigens by DCs (22–24). Although various DC-based cancer vaccines have been exploited in the past and the properties, advantages, and disadvantages of each extensively reviewed (20, 26, 27), it is most commonly DCs cultured *ex vivo* from peripheral blood monocytes (monocyte-derived dendritic cells, moDCs) that have shown the greatest clinical benefit. Indeed, one such vaccine, Provenge, has entered the market for the routine treatment of prostate cancer and involves the co-culture of moDCs obtained by leukapheresis, with the TSA, prostatic acid phosphatase (PAP), to which T-cell responses have been detected following their reinfusion into patients (28).

The majority of the clinical trials involving cancer vaccination have used autologous moDCs cultured *ex vivo* and pulsed with soluble TAA before re-administration to patients with the hope of inducing a tumor-specific immune response (29). Although many of these trials have shown that immunotherapy based on the use of mature moDC is safe, well-tolerated, and able to elicit an immune response against the tumor (27), the overall results have been disappointing, showing significant inter-trial variability between outcomes (30–32). This may be due, in no small part, to the donor-to-donor variability in yield and quality of moDCs, which is further compounded by long-term exposure to chemotherapeutic agents. Furthermore, the poor capacity of moDCs to cross present exogenous TAAs to CTLs, limits the magnitude of the cell-mediated immunity required to clear established tumors.

The recent identification of cross presenting DCs in man, equivalent to the CD8α^+^ subset that has long been recognized in the mouse, has rejuvenated interest in the use of DCs in cancer immunotherapy (33, 34). These cells are defined by their expression of the surface marker CD141 and the chemokine receptor XCR1 and are found in the peripheral blood, tonsils, and bone marrow (35). They display an unrivaled capacity to cross present exogenous antigen to CD8^+^ T cells, and hence to elicit effective cell-mediated immunity. While the properties of CD141^+^ XCR1^+^ DCs make them ideal candidates for immunotherapy against cancer, their trace numbers in peripheral blood (<0.1%) limit their therapeutic exploitation (25). Alternative sources of cross presenting DCs, including their isolation from the spleen or *in vitro* differentiation from hematopoietic progenitor cells, have so far failed to overcome these obstacles (36).

Since the holy grail of cancer immunotherapy is to stimulate tumor-specific T cells that will elicit a cytotoxic response with high specificity and minimal toxicity, adoptive transfer of TAA-specific T cells has gained popularity over the past few years (37, 38). Adoptive T-cell transfer involves the isolation of T lymphocytes from the patients and their reinfusion to treat disease. The adoptive transfer of T cells was first documented in rodents in 1955, where it was noted that T cells obtained from lymph nodes draining a tumor were able to confer immunity when transferred into the peritoneum of a secondary host, bearing a similar tumor (39). Almost three decades later, it was observed that the incubation of murine splenocytes with IL-2 generated large numbers of cells, called lymphokine-activated killer (LAK) cells, which were capable of lysing tumor cells with little effect on other somatic cells (40). These LAK cells were later shown to decrease tumor number and size in humans in a wide variety of tumors including pulmonary and hepatic metastases (41, 42). This work served as the basis for the use of tumor-infiltrating lymphocytes (TILs) in immunotherapy (43, 44). The combination of a lymphodepleting preparative regimen with adoptive transfer of TILs and administration of IL-2 has been shown to promote cancer regression in patients with metastatic melanoma, leukemias, and other types of tumor (44, 45).

The possibility of genetic modification of the T cells to overcome the immunosuppressive environment created by the tumor may lead to more effective therapies in the future, although current strategies for genetic modification are limited (46) and T cells are known to constitute particularly intractable targets. Nevertheless, the possibility of genetically engineering T cells to recognize specific TAAs makes it possible to target potentially any tumor using adoptive T-cell transfer (47), while leaving other tissues intact. The majority of clinical approaches use virus-based transduction of tumor antigen-specific T-cell receptor (TCRs) or chimeric antigen receptors (CARs) to generate T cells stably expressing tumor-specific transgenes which, although efficient, is expensive and risks insertional mutagenesis. Non-viral approaches to genetically engineer T cells have so far utilized transposon elements such as piggyback or zinc finger nucleases (46). TALEN and CRISPR/Cas-9-based-approaches, which allow for the insertion of transgenes into defined chromosomal loci, are, however, currently being actively explored (48, 49). Despite the attractiveness of using CAR technology to target cancer, only the treatment of B cell leukemia has so far proven successful using this approach. Furthermore, recent work has shown that the treatment of patients with myeloma or melanoma using T cells engineered to express affinity-enhanced TCRs for an HLA-A*01-restricted epitope of MAGE-A3 resulted in severe myocardial damage secondary to widespread T-cell infiltration leading ultimately to fatal cardiogenic shock. These findings have clearly shown how even altering the affinity of the TCR for its ligand may introduce unanticipated cross-reactivity with potentially fatal off-target toxicity (50).

Although much effort has been invested into the adoptive transfer of unmodified T cells in the treatment of cancer, outcomes have been disappointing. It is, for instance, sometimes challenging to identify tumor-specific T cells in patients with non-solid tumors. TILs can also be difficult to isolate from biopsies of most melanomas. *Ex vivo* expansion of tumor-specific CTLs can also prove difficult: in the case of EBV-specific CTLs, for instance, 3 months is required for the production of sufficient CTLs for re-administration to the patient, with obvious implications for disease progression. Often, reinfusion of T cells is required following adoptive transfer for the induction of a durable response due to the exhaustion of the expanded CTLs.

NK cells have likewise been used for adoptive transfer, due to their innate ability to recognize tumor cells deficient in MHC class I. NK cells have been isolated form peripheral blood, expanded *ex vivo*, activated using IL-2 or, more recently, the combination of IL-12, IL-15, and IL-18 (51), and re-administered to patients (52). Interestingly, although the use of autologous cells is normally preferred, several studies have demonstrated that the use of allogeneic NK cells is significantly more effective (53, 54). Accordingly, the results of several studies have shown NK cells to be well-tolerated following adoptive transfer with encouraging results of up to 20 months’ survival following their administration (55). Nevertheless, low circulating numbers of NK cells in peripheral blood, coupled with the difficulty in their expansion and their inability to stimulate a robust response *in vivo*, limits their use in immunotherapy.

Given the difficulty of obtaining sufficient numbers of cells to target tumors *in vivo*, the advent of induced pluripotency offers unrivaled opportunities. The proven ability to produce iPS cells from individuals in a patient-specific manner with the capacity for indefinite self-renewal and unrestricted differentiation potential, may facilitate the scale-up in production of critical hematopoietic cell types, for many of which, protocols have already been optimized. Below, we outline the history of induced pluripotency and discuss the properties that make them attractive candidates for use in immunotherapy.

## Brief History of Pluripotency

Since their first description, embryonic stem (ES) cells have been regarded as the “gold standard” for pluripotency, displaying the capacity for indefinite self-renewal and differentiation into any somatic cell type, irrespective of its embryonic germ-layer of origin. Mouse ES cells were first isolated in 1981 by Martin Evans (56), work which later earned him the 2007 Nobel Prize for Physiology or Medicine. Nevertheless, it was not until 1998 that Thomson and colleagues succeeded in deriving ES cells from the inner cell mass of human blastocysts that were surplus to requirements following *in vitro* fertilization (57). Human ES cell lines, like their mouse counterparts, were found to be pluripotent, expressing embryonic markers such as SSEA-3, SSEA-4, TRA-1–60, and alkaline phosphatase and, following injection into immune compromised mice, forming teratomas containing cell types and tissues from all three embryonic germ layers.

Since their first derivation, there has been much interest in the use of human ES cells as a source of diverse cell types for drug discovery, regenerative medicine, and immunotherapy. However, their use has been highly controversial due to the ethical sensitivities surrounding their derivation from human blastocysts, as well as the inevitable scientific constraints of using an allogeneic source of cells. In 2006, Yamanaka and colleagues demonstrated the feasibility of deriving pluripotent stem cells from adult mouse fibroblasts by retroviral transduction with genes encoding Oct3/4, Sox2, c-Myc, and Klf4 (58). These so-called iPS cells are indistinguishable at the cellular level from conventional ES cells, acquiring the capacity for indefinite self-renewal, unrestricted differentiation potential and, following injection into mouse blastocysts, the ability to generate germline-competent chimeras. These findings were subsequently translated to human dermal fibroblasts in 2007 by two independent groups (59, 60), showing, in principle, the feasibility of generating iPS cells on an individual basis. This seminal work offered a means of “personalizing” pluripotency in a manner free of the ethical concerns, while simultaneously addressing the immunological issues that limit the effectiveness of allogeneic therapies. Indeed, the production of iPS cells in an autologous fashion has paved the way for harnessing the potential of pluripotency for immunotherapeutic intervention in the pursuit of treatments for numerous indications.

Given the broad clinical applicability that iPS cells may enjoy in the future, there have been many efforts to develop and optimize the re-programming process to increase the safety profile of the resulting cell lines (Table 1). Protocols based on retroviral transduction may result in insertional mutagenesis while inducing the ectopic upregulation of developmental genes, which may subsequently render cells immunogenic (61). The direct delivery of re-programming proteins into somatic cells (62) and transfection with synthetic mRNA (63) have both proven successful, albeit yielding iPS cells at very low efficiency. Interestingly, small molecules, such as the histone deacetylase inhibitor, valproic acid, have been demonstrated to increase this efficiency by up to 100-fold (64). More importantly, recent work has shown that full re-programming may be achieved with a combination of seven small molecules alone, suggesting that induced pluripotency may not be dependent on the use of virus-based delivery systems (65). To achieve this, the authors screened 10,000 small molecules in order to find suitable replacements for each transcription factor. Three molecules, forskolin, 2-methyl-5-hydroxytryptamine, and D4476 were, for instance, identified as chemical substitutes for Oct3/4.

**Table 1:** **Methods of reprogramming and complications associated with derived iPS cell lines**.

	Advantages	Disadvantages
Forced expression of genes via retrovirus	Well-characterized method, long history of use, arguably a simple approach and low cost, relatively high reprogramming rates of 0.01–0.02%	Integration into the genome may generate immunogenic cells, virus will only enter cells in mitosis, use of oncogenes such as c-Myc
Small molecules	Low cost of compounds, increases the efficiency of reprogramming	Only recent reports of full reprogramming achievable with small molecules alone: further characterization of lines generated needed
Synthetic miRNA	No integration within the genome	Very low reprogramming efficiency, miRNA degrades rapidly, modification of miRNA complicated, and time-consuming
Forced expression of genes via Sendai virus	No integration into the genome, higher efficiency of reprogramming than using retrovirus, diluted out of culture upon passage rapidly, high reprogramming rate of 0.1%	Difficult to work with, therefore most commonly used as pre-packaged “kits,” which are expensive compared to other viral methods of reprogramming
Episomal plasmid vector system	No genomic footprint	Very low efficiency of reprogramming (0.0002%), loss of episomal plasmid
Stimulus-triggered acquisition of pluripotency (STAP)	No nuclear transfer or introduction of transcription factors	Limited capacity for self-renewal when compared to ES cells.
		Reports have yet to be independently verified

Perhaps the most dramatic advance in this rapidly evolving field has, however, been the recent description of stimulus-triggered fate conversion of cells (66), in which transient exposure of terminally differentiated cells to adverse conditions such as low pH, induces the upregulation of pluripotency genes. This approach has been shown to confer on cells such as murine lymphocytes, the capacity to form germline-competent chimeras following injection into recipient blastocysts, or the formation of entire offspring in tetraploid complementation assays. However, this method has yet to be verified independently and further characterization of the iPS cell lines produced in this way must be conducted and the translation of protocols to adult human cells has yet to be achieved, this approach may one day allow the generation of iPS cells lines with minimal intervention, compatible with downstream clinical applications.

**Figure 1:**
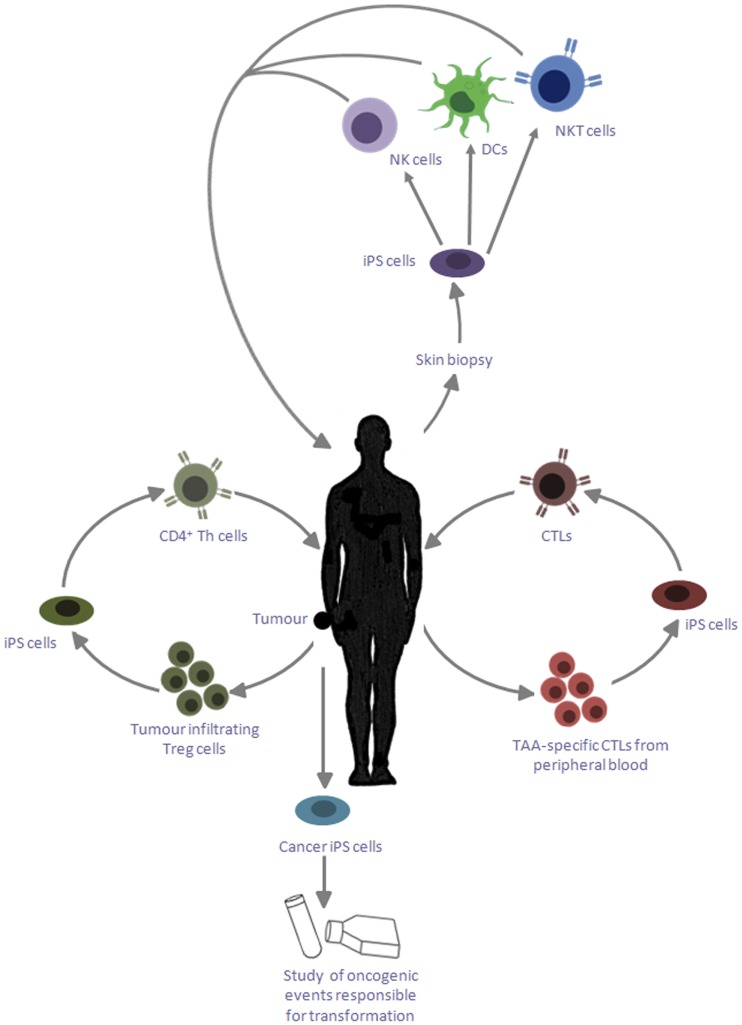
**Applications of iPS cells for cancer immunotherapy**. iPS cells reprogramed from skin biopsies of cancer patients can be differentiated into DCs, which can be reintroduced into patients to cross present TAA. These iPS cells can also be differentiated into NK cells and NKT cells, which can be adoptively transferred into the patient to target cancer cells; iPS cells can be generated by reprogramming tumor-specific CTLs, which can provide an unlimited source of naïve CD8^+^ T cells with the desired specificity; tumor-infiltrating Treg cells may likewise be reprogramed into iPS cells and redifferentiated into CD4^+^ Th cells, which are capable of providing help to the CTLs to target cancerous cells; these iPS cells can also be exploited to study the genetic basis of transformation and its influence of primary cell types.

Although traditionally much of the interest in iPS cells has focused on applications in regenerative medicine, other indications include their use as a novel source of hematopoietic cell types for cancer immunotherapy (Figure 1). The opportunity to derive iPS cells in a patient-specific manner, together with their tractability for genome editing using newly developed technologies such as the CRISPR–Cas-9 system (67) make them attractive candidates for such applications. Furthermore, their indefinite capacity for self-renewal may greatly facilitate the scale-up of cell production, offering unrivaled opportunities for overcoming many of the obstacles encountered using conventional sources of cells.

## Exploiting Induced Pluripotency for the Study and Treatment of Cancer

### Cancer iPS cells as models of disease progression

The use of iPS cell lines to model *in vitro* a broad range of human disease states has already begun to yield important advances in our understanding of their pathogenesis and progression. Nevertheless, the generation of iPS cells from primary cancer cells has remained a significant challenge, proving successful for only a limited number of cancers due, most likely, to the associated genetic or chromosomal abnormalities introducing a state of genetic instability (68). Although reprogramming of gastrointestinal cancer cells to a pluripotent state has been achieved by careful modification of culture conditions and re-programming factors (69), it has proven necessary to use retroviral vectors to introduce the necessary transgenes, which risks the introduction of confounding mutations that may interfere with the phenotype of cells differentiated from the resulting iPS cell lines. Application of the latest non-viral reprogramming technologies to primary cancer cells is, therefore, paramount for gaining insight into the impact that oncogenic events may have on a range of primary cell types. The potential that such an approach offers for drug discovery and toxicity screening may facilitate the future identification of therapeutic targets as well as novel neo-antigens that may be exploited for vaccination purposes.

### Cancer vaccination using iPS cell-derived DCs

Given the significant donor-to-donor variability encountered in the use of moDCs for cancer immunotherapy, early research focused on the potential of pluripotency to provide a more homogenous source of DCs amenable to scale-up. Accordingly, several groups reported the successful differentiation of functional DCs from both mouse and human ES cells (70, 71). Since the use of animal products for their differentiation made them unsuitable for clinical applications, Tseng and colleagues succeeded in developing protocols for their differentiation in an animal product-free manner, compatible with their downstream use *in vivo* (72). Although these DCs were shown to be functional, possessing the ability to endocytose, process, and present foreign antigen to naïve CD4^+^ T cells, their clinical utility was restricted both by their limited capacity for cross presentation of antigen to MHC class I-restricted CD8^+^ T cells and their differentiation from allogeneic sources, requiring matching at certain HLA loci. Recent work in our laboratory has, however, demonstrated that CD141^+^XCR1^+^ DCs can be successfully derived from human iPS cells using a cocktail of GM-CSF, stem cell factor (SCF), vascular endothelial growth factor (VEGF), and bone morphogenetic protein-4 (BMP4). This protocol was found to be compatible with the exclusion of all animal products, ensuring the downstream clinical compliance of the DCs obtained (73). Unlike moDCs used for comparison, these DCs were shown to efficiently cross present the TAA, Melan A, supplied exogenously in recombinant form, to naïve CD8^+^ T cells *in vitro*, stimulating a primary Melan A-specific immune response that could be tracked using tetramer technology (73). Since iPS cells have indefinite capacity for self-renewal *in vitro*, this approach provides a potentially unlimited source of autologous DCs that might bypass the issue of patient-to-patient variability and the confounding effects of long-term chemotherapy that impacts adversely on the circulating monocytes from which conventional moDCs are derived.

### Differentiation of T cells for adoptive transfer

While the use of DCs to stimulate TAA-specific immune responses *in vivo* offers the prospect of establishing durable immunological memory, an alternative strategy for cancer immunotherapy has been the adoptive transfer of antigen-specific T cells, expanded *ex vivo*. Since such expansion regimes risk the functional exhaustion of the resulting cells, the differentiation of potentially unlimited numbers of primary T cells from pluripotent stem cells has proven an attractive goal. The co-culture of mouse ES cells with the OP9 stromal cell line constitutively expressing delta-like ligand 1 (OP9-DL1), has been shown to successfully support their differentiation into T-cell progenitors (74, 75). Nevertheless, their final commitment to the T-cell lineage requires their introduction into fetal thymus organ cultures, so as to provide a microenvironment conducive to TCR gene rearrangement and subsequent positive selection of a diverse CD4^+^ and CD8^+^ T-cell repertoire. On transferring these ES cell-derived T cells into RAG2^−/−^ mice, immune reconstitution was readily observed, strongly suggesting that T cells generated in this way were functionally mature. Although these findings suggest that pluripotent stem cells may serve as a potentially unlimited source of naïve T cells for adoptive transfer, the requirement for an appropriate thymic microenvironment to support V(D)J recombination and positive selection poses significant ethical and pragmatic barriers to the translation of protocols to the human.

A logical way to overcome this hurdle might be to harness induced pluripotency to generate iPS cells from T cells that have already undergone V(D)J recombination and are known to exhibit a desirable antigen specificity. Any T cells differentiated from the parent iPS cell line would maintain the original antigen specificity of the parent cells, and may, therefore, differentiate *in vitro* in a thymus-independent manner. Recent studies have reported the successful differentiation of antigen-specific T cells from an iPS cell line itself generated from CTL specific for an epitope from the melanoma antigen MART-1 (76). These cells were expanded by stimulation with anti-CD3 mAbs, thereby generating CD8^+^ T cells, which were shown to respond to MART-1, demonstrating retention of their original antigen specificity.

Given the low frequency of tumor-specific T cells in the periphery of individuals and difficulties surrounding their identification, Themeli and colleagues exploited the tractability of iPS cells for genetic modification to introduce a bicistronic lentiviral vector encoding 19–28z, a CAR specific for CD19, expressed by the majority of leukemias and lymphomas. The authors were able to optimize differentiation conditions to allow for serum and feeder free generation of hematopoietic progenitor cells which, when co-cultured with OP9-DL1 stromal cells in the presence of SCF, Flt3L, and interleukin-7 (IL-7), differentiated into T cells expressing the CD19-specific CAR. T cells produced in this way were activated by CD19^+^ APCs and, upon infusion into mice, potently inhibited tumor progression (77).

While the use of CARs may circumvent the requirement for the identification of antigen-specific T cells, an alternative method of capturing desirable antigen specificities might be to exploit TILs whose presence within a developing tumor provides compelling evidence for their specificity. Whereas CD8^+^ CTL are readily obtained from tumor biopsies and lend themselves to reprogramming, other T-cell subsets are also evident including regulatory T (Treg) cells. The presence of these Treg cells is known to negatively correlate with survival rates (78), due to their capacity to suppress anti-tumor immune responses and facilitate evasion of the developing tumor from the host immune system. If tumor-specific Treg cells could likewise be reprogramed to pluripotency, their redifferentiation along the T-cell lineage might provide opportunities for their phenotypic reassignment into effector T cells, providing a valuable source of CD4^+^ T-cell help for endogenous CTL responses in danger of exhaustion.

One of the major hurdles to harnessing this approach is defining extracellular Treg-specific markers. Currently, the most widely used marker for Treg cells is Foxp3 among CD4^+^CD25^+^ cells (79). As this transcription factor is expressed solely in the nucleus, however, sorting of cells based on its expression is not feasible. Nevertheless, recent work has demonstrated that CD127 expression inversely correlates with Foxp3 and hence the suppressive function of human CD4^+^ Treg cells (80). In addition to low CD127 expression, expression of CD45RA is also apparent in human CD4^+^ Treg cells (81): sorting cells based on a CD4^+^CD25^+^ CD127^low^CD45RA^+^ phenotype would, therefore, represent the most effective strategy currently available for isolating antigen-specific Treg cells infiltrating the tumor microenvironment.

### NK cell-based immunotherapy

Although much interest has focused on the use of NK cells in cancer immunotherapy, obtaining sufficient numbers for administration to patients remains a significant limitation. In 2005, Woll and colleagues used a two-step process to differentiate human ES cells into NK cells *in vitro*. These cells had the ability to lyse human tumor cells deficient in MHC class I expression and up-regulate cytokine production (82). Subsequently, NK cells were successfully differentiated from human iPS cells, using a similar two-stage culture system (83), the cells obtained representing a pure population that did not require cell sorting or co-culture with xenogeneic stromal cells. Moreover, sufficient cytotoxic NK cells could be differentiated from 250,000 iPS cells to treat a single patient, suggesting that iPS cells provide a scalable platform for the clinical implementation of such an approach.

In addition to *bona fide* NK cells, it has recently proven possible to derive NKT cells from iPS cells. NKT cells are characterized by the expression of an invariant TCR encoded by Vα24–Jα18 in humans and Vα14–Jα18 in mice (84). These cells share the properties of both NK cells and T cells and are thought to play an important role in cancer immune surveillance (85). Indeed, NKT cells differentiated from mouse iPS cells were shown to secrete large quantities of IFNγ and actively suppress tumor growth *in vivo* (86). The differentiation of NKT cells from iPS cells may, therefore, make this elusive cell type readily accessible for cancer immunotherapy in the future.

## Conclusion

Although significant advances have been made in cancer immunotherapy over the past decade with the discovery of human cross presenting DCs and the use of CARs and TCR transfer for the generation of more effective T-cell therapy, the requirements for high specificity, minimal toxicity, and the capacity for immunological memory have yet to be achieved. It has been suggested that since no single therapy is likely to fulfill all these criteria, adoptive transfer of tumor-specific T cells might be combined with DC vaccination to generate a durable immune response (87). Given the unrestricted differentiation potential of iPS cells, prospects for the differentiation of either cell type from the same patient-specific cell line provide a comprehensive approach. Furthermore, their potential for the efficient scale-up of cell production and tractability for new generation genome engineering tools, such as the CRISPR/Cas-9 system and transcription activator-like effector nucleases (TALENs) (48, 49) may herald a new era in cancer immunotherapy, in which treatments are exquisitely tailored to the individual needs of the patient.

## Conflict of Interest Statement

The authors declare that the research was conducted in the absence of any commercial or financial relationships that could be construed as a potential conflict of interest.
